# Assessing the preparedness and feasibility of an e-learning pilot project for university level health trainees in Ghana: a cross-sectional descriptive survey

**DOI:** 10.1186/s12909-020-02380-2

**Published:** 2020-11-25

**Authors:** Robert Kaba Alhassan

**Affiliations:** Institute of Health Research, University of Health and Allied Sciences, Ho, PMB 31 Ho, Ghana

**Keywords:** E-learning, Feasibility, Ghana, Health trainees, Preparedness, Innovation, Nursing and midwifery, Policy implications

## Abstract

**Background:**

Ghana is challenged with shortage of critical human resources for health particularly nurses and midwives in rural hard-to-reach communities. This shortage potentially hinders efforts towards attaining universal access to basic healthcare. More importantly, poor quality of pre-service training for health trainees has the potential to worsen this predicament. There is therefore the need to leverage emerging digital innovations like e-learning to complement existing efforts. This study was conducted several months before the outbreak of COVID-19 to investigate the preparedness, acceptability and feasibility e-learning innovation for nursing and midwifery trainees.

**Methods:**

The study is a cross-sectional descriptive survey involving nursing and midwifery students (*n* = 233) in one of Ghana’s public universities, located in the Volta region of Ghana. Simple random sampling technique was used to collect responses from eligible respondents using a structured questionnaire. Descriptive statistical analysis was done using STATA software (version 12.0).

**Results:**

It was found that nearly 100% of respondents owned smartphones that were used mostly for learning purposes including sharing of academic information. Over 70% of respondents particularly used social media, social networking applications and internet searches for learning purposes. Health trainees were however constrained by low bandwidth and lack of seamless internet connectivity within their learning environments to maximize the full benefits of these e-learning opportunities.

**Conclusion:**

Respondents were predominantly prepared for an e-learning pilot project. These feability findings suggest e-learning is a huge potential that can be used to augment existing approaches for pre-service training of health trainees in Ghana, when implementation threats are sufficiently addressed. Compelling findings of this study are therefore timely to inform evidence-based policy decisions on innovative digitial solutions for pre-service training of health workforce even as the world adapts to the "new normal" situation induced by COVID-19.

## Background

There is wide recognition that the recent, rapid extension of mobile, Information Communication Technology (ICT) and other digital innovations in sub-Saharan Africa (SSA) offer huge opportunities for achieving sustained improvements in frontline health worker training. According to an evaluation done in 2015, the world had a total of 7 billion mobile phone lines or numbers [1]. At the end of 2016, Ghana had a mobile phone subscription totaling 38.31 million [1]. Additionally, Ghana currently has the highest mobile phone penetration in West Africa [1] with mobile adoption rate of 55%, higher than the regional average of 45% [2]. Likewise, by the third quarter of 2019, Ghana’s unique mobile subscribers were 16.7 million while smartphone devices and mobile phone internet users were 15.1 million and 10.7 million respectively [2].

According to a World Health Organization (WHO) report [3], Ghana is among African countries suffering from critical shortage of frontline health staff including nurses and midwives [4]. The consequent effect of these limited frontline health staff on quality of healthcare delivery are not farfetched. In rural parts of SSA, frontline health staff shortage relates largely to nurses and midwives, who are the primary source of healthcare since medical doctors are few and will remain so in the foreseeable future. The capability of nurses and midwives in rural areas to provide high-quality healthcare thus remain a determinant of a country’s health sector performance [5, 6]. Unfortunately, rural and peripheral regions such as Volta region are the most deprived and underserved when it comes to the quantity and quality frontline health workers [7].

Over the years, Ghana has witnessed unprecedented increases in nursing and midwifery trainee enrolments to bridge the staffing deficits in the country [6, 7]. Nonetheless, these increases are a potential threat to the quality of pre-service training due to an overwhelmed physical infrastructure for training schools coupled with inadequate teaching staff, learning materials such as textbooks and clinical mannequins for simulations [8, 9].

In light of these constraints, digital innovations like e-learning are increasingly embraced by countries across the globe to enhance the quality of pre-service training for frontline health staff [8]. Digital innovations such as e-learning have the potential to create a learner-centered environment that is interactive, collaborative, situational and learner-relevant [10]. Traditional teacher-centered learning conversely situates the teacher in an active role while students take a more passive or receptive role [11–18].

Digital innovations such as e-learning allow for a shared space for groups to interact in ways not known before such as Wikis, Blogs and group Live Chats. Digital innovations like e-learning can therefore be a highly effective method in nursing and midwifery education in developing countries like Ghana [19]. Moreover, empirical studies suggest that successful implementation of e-learning projects depends strongly on the design of such interventions. E-learning innovations are more likely to succeed when they are adapted to the needs and preferences of the various target user groups, embedded in established social practices [20].

**Fig. 1:**
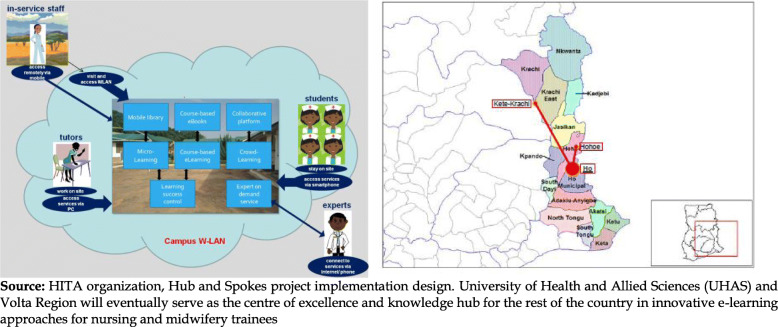
Planned hub and spokes approach to implementing the e-learning pilot project

This paper reports on the outcome of an exploratory study on the feasibility of a digital innovative e-learning pilot project for frontline health trainees in one of Ghana’s public universities. An illustration of the planned e-learning pilot project is shown in Fig. 1. The study explored preparedness and feasibility conditions among students in terms of mobile phone take-up and patterns of use among students; use of existing e-learning applications; social media and networks for learning purposes.

## Methods

### Study design

This is a cross-sectional descriptive survey conducted to determine the preparedness and feasibility of implementing a pilot e-learning project. The study design is deemed appropriate because it promotes coverage of larger sample size and enhances generalizability of the findings to similar study populations. The design also enables the researchers to describe and examine associations between key variables of interest.

### Study setting and population

The study was conducted in a public university solely dedicated to the training of healthcare professionals (name withheld to maintain anonymity) The university is located in the Volta region. The Volta region is one of the 16 administrative regions in Ghana and it lies on the eastern side of the country. The region shares geographic boundaries with the Republic of Togo to the east; the Greater Accra, Eastern and Brong-Ahafo regions to the west; the Oti region to the north and the Gulf of Guinea to the south. The region has a total land area of 20,570 square kilometers, representing approximately 9% of the total land area of Ghana. According to the 2010 Population and Housing Census (PHC), the Volta region share of the estimated 30 million Ghanaians was 8.6%.

At the time of conducting this study, the university had a population of about 5,000 students and about 600 teaching and non-teaching staff. The University has six schools including the School of Nursing and Midwifery. This school is the most populous school constituting over 50% of the entire university population and about 30 teaching and non-teaching staff. The nursing and midwifery school has three departments namely: Nursing, Midwifery and Public Health Nursing. The school offers three streams of programs: regular (for high school graduates), Sandwich (for practicing nursing staff with auxiliary certificates) and Top-up (for practicing nursing staff with professional certificates).

#### Sampling and sample size

The study randomly recruited undergraduate students (henceforth called health trainees) of the nursing and midwifery school because of the focus on nursing and midwifery pre-service training. Total list of eligible students was obtained from all three departments (General Nursing, Midwifery and Public Health Nursing) across all the four years of study (i.e. years 1 – 4). Determination of the sample size was based on Krejcie and Morgan [21] statistical tool for determining sample size based on known population. This statistical tool gives the recommended sample size for a finite population at 95% level of confidence. Based on Krejcie and Morgan [21] formula, the sample size of 300 was deemed representative of the estimated student population of about 3,000 in the nursing and midwifery school. Moreover, the sample size of 300 accounted for a 5% non-response rate.

### Inclusion and exclusion criteria

Eligible respondents were health trainees aged 18 years and above; pursuing their studies in the school of nursing and midwifery and finally respondents who voluntarily consented to participate. Exclusion criteria included health trainees from other schools apart from the school of nursing and midwifery, and health trainees who were not registered for the academic at the time of conducting this study (i.e. health trainees on leave of absence or deferment of their programme of study).

### Data collection tool and piloting

Structured questionnaire was developed to ascertain preparedness, acceptability, adaptability and feasibility of an e-learning pilot project for nursing and midwifery trainees. The development of the data collection was based on a grounded theory approach [22] where previous qualitative engagements with the target respondents generated thematic areas on feasibility of a pilot e-learning project. Thus, the survey tool was developed based on findings from these earlier qualitative face-to-face interviews but findings of these qualitative studies are yet to be published. Additionally, field visits were done to other nursing and midwifery schools in six other regions in Ghana in 2011, 2016 and 2017 and 2016 in Zambia. The grounded theory approach is an acceptable strategy for developing trustworthy data collection tools which have not necessarily undergone psychometric testing [22].

Additionally, to promote internal validity and reliability of questionnaire items, Cronbach’s alpha was checked and the scale reliability coefficient was found to be above the 0.80 rule of thumb [23]. Also, the questionnaires development was guided by the research objectives followed by piloting among students with similar characteristics of the study participants. The pilot showed that all questions were understood as intended by the researchers without ambiguity. Independent peer-review and validation of the questionnaire was also done by external reviewers to promote internal validity of the tool.

The questionnaire comprised of eight sections namely: Section A (background information of respondents); Section B (Preparedness Proxy 1: Mobile Phone Ownership and Usage of Applications); Section C (Preparedness Proxy 2: Use of Mobile Phones and Related Devices for Education/Academic Purposes); Section D (Preparedness Proxy 3: Rated Satisfaction with Current University ICT Infrastructure (Computer Laboratory)); Section E (Preparedness Proxy 4: Rated Satisfaction with Current University ICT Infrastructure (Internet)); Section F (Preparedness Proxy 5: Rated Preparedness of Teaching Faculty for e-Learning); Section G (Preparedness Proxy 6: Rated Preparedness of ICT Staff for e-Learning); Section H (Suggestions towards Successful Introduction of e-Learning Programme in the University).

### Data collection

The study was conducted between 24^th^ July and 8^th^ August 2017, by administering 300 paper questionnaires to the eligible respondents at random from the list of students in the School’s register. Next, codes were assigned to all target students of the School. Written pieces of paper were randomly picked (without replacement) by a blinded research assistant. Subsequently, the randomly sampled students were contacted via phone by research assistants and questionnaire administered to those who voluntarily agreed to participate. Students who refused to participate were replaced by randomly picking a replacement from the pool of eligible respondents in the school’s register.

### Data analysis

Data were first captured onto Microsoft Excel Spreadsheet, cleaned and coded before analysis with STATA (version 12). Descriptive statistical analyses (means, frequencies and percentages) were run to compare relevant variables of interest. Pictural representation of the data was also presented in bar charts as appropriate. Statistical significance was determined at 95% confidence level.

## Results

### Background information

In total, 300 structured questionnaires were administered to respondents out of which 233 were correctly completed and returned, representing a response rate of 78%. It was found that one in two respondents were enrolled in a nursing course for either 2-years, 3-years or 4- years. Also, 42% of the respondents were pursuing a midwifery course, and 9% were in a public health nursing programme. Three in four respondents were females. In terms of age distribution, respondents’ ages ranged from a minimum of 20 years to an upper limit of 45 years. The mean age was approximately 30 years (see Table 1).

**Table 1:** Background information of respondents

Characteristics	Frequency (f)	Percentage (%)
**Gender (*n*** **= 233)**		
Male	58	25
Female	175	75
**Programme (*n*** **= 233)**		
Nursing	114	49
Midwifery	98	42
Public Health Nursing	21	9
**Age (*n*** **= 233)**	**Mean**	**[95% CI]**
	30	[20 45]

Source: Field Data (2018); Legend: *CI* Confidence Interval

### Ownership and usage of smartphones and applications

**Fig. 2:**
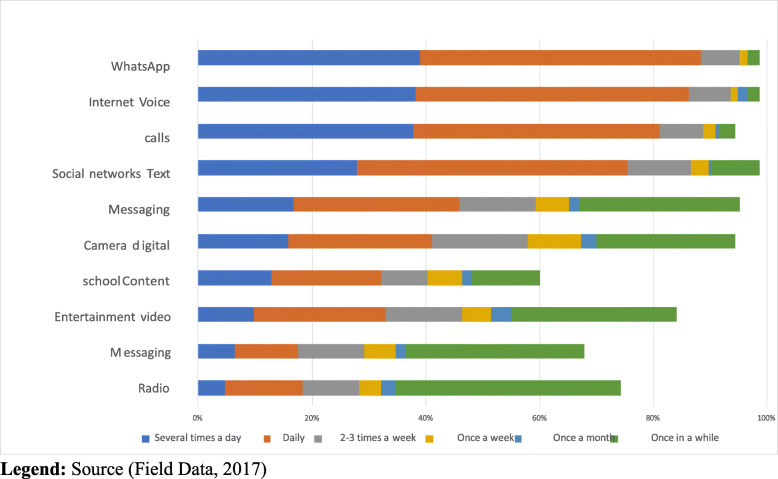
Duration of smartphone usage per day (*n* = 233)

**Fig. 3:**
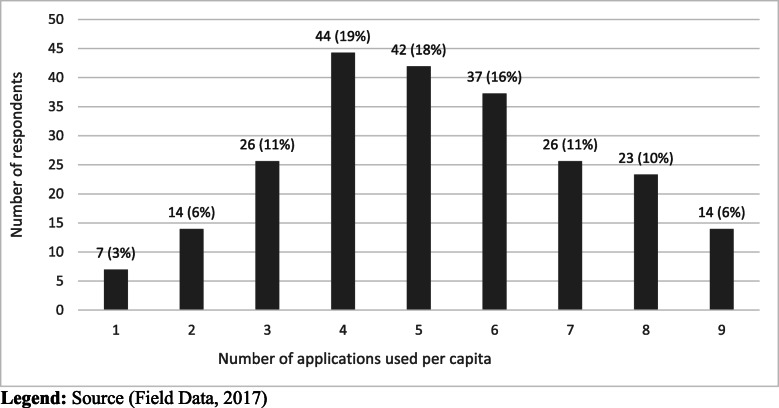
Mobile phone applications used by respondents (*n* = 233)

All 233 respondents stated that they owned a smartphone; the most common brands owned were Samsung, Techno, Infinix, and ITEL; it was found that WhatsApping was the most frequently used function of smartphones by respondents on daily basis followed internet voice, phone calls, social network texting and short messaging services (SMS). The least used function was listening to radio (see Fig 2). Also, it was discovered that 18% of respondents used their smartphone for 20 minutes or less per day, whereas 11% used their phones for 21-30 minutes. Also, it was observed that 35% of respondents used their smartphones for 31-60 minutes and 36% used their phones for more than one hour in a day. Additionally, 61% of the respondents used at least five applications each day (see Figure 3).

The results further showed that 88% of the respondents used *WhatsApp* on daily basis. Additionally, 86% of the respondents used their phones to access the internet on daily basis. Social media networks such as Facebook was mentioned by 76% of the respondents. Out of the 233 respondents, 60% of them mentioned they used existing “e-learning school content”.

### Use of smartphone applications

Moreover, the results showed that “searching for information on the internet” was the most widely and frequently used purpose of smartphones and their applications, followed by “reading”. The interviews also revealed that lecturers often asked students to obtain complementary learning materials such as YouTube videos from the internet. However, the respondents indicated that no specific websites were given to help find adequate and relevant sources of information on subject areas. The shortcoming of this practice, as reported by students was that the quality of the online material was not assessed and double checked beforehand by lecturers.

Approximately 80% of the students used the smartphones for “WhatsApp chatting” and “social networking” (i.e. creation of learning groups). However, it was found that these usage patterns or behaviours were largely informal. Respondents specifically mentioned lack of established structures in the university to fully integrate such digital e-learning innovation tools in the routine teaching and learning practices.

**Fig. 4:**
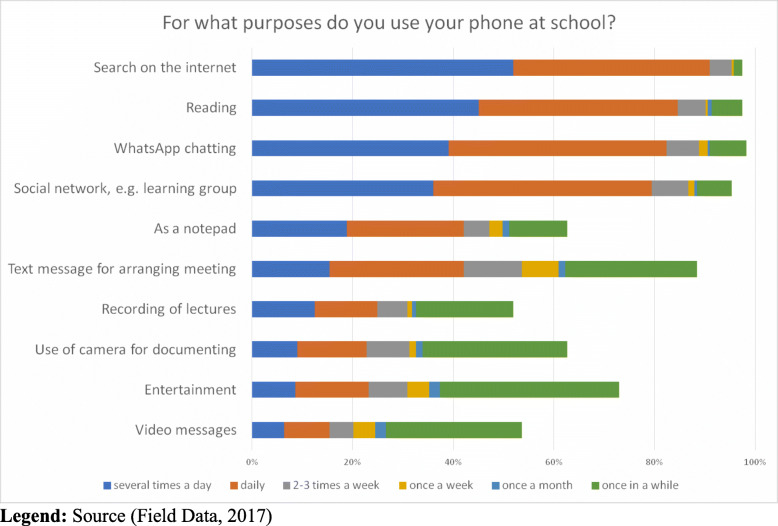
Education related uses of the phone (*n* = 233)

Moreover, it was found that the use of smartphones for recording lectures or documentation purposes was not widespread. Barely 33% of the respondents said they utilize this e-learning function of the smartphone. The types of phones used by many respondents did not have cameras that allowed them to take pictures and make audio recordings that are of sufficient quality for learning purposes (see Figure 4).

Additionally, it was found that 78% of the respondents said the smartphone they used was either “very good” or “good” for communicating with parents, relatives or friends. Furthermore, use of the smartphones for “learning” was rated as either “very good” or “good” by 74% of the students. Use of smartphones to network with other students was rated as either “very good” or “good” by 65% of the respondents; 46% of the respondents considered the smartphone as “very good” or “good” for “communicating with lecturers” and “working offline” including making lecture notes (42%).

The findings also revealed a significant variation in the average monthly expenditure on mobile charges; 21% of respondents said they spent approximately €1.80 per month; 25% spend between € 2.00 - 3.60; 21% spent between € 3,78 - 5.40; 13% spent between € 5.58 -7.20; 9% spent between € 7.37 - 9.00 and 11% spent more than €9.

**Fig. 5:**
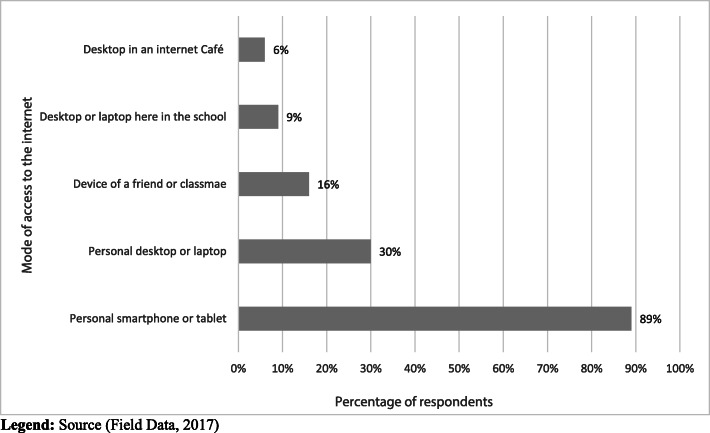
Applications used on the phone (*n* = 233)

Results on internet access showed that 50% of respondents said they accessed the internet “several times” per day; 42% said they do so “at least once” per day. Only three respondents stated they did not access the internet at all. It was observed that the most common means to access the internet by students was by their own smartphones or tablets (89%). Nearly a third of the respondents said they used their personal computers (i.e. desktops or laptops). Even though the university is equipped with a computer laboratory, only 9% of respondents used school-owned computers to access the internet (see Figure 5).

It was also discovered that over 50% of respondents were from middle-income households, although there was substantial variation in this respect. The average personal budget students had per month was about €36.00^1^. Some students also reported complementing monthly remittance from parents/guardians with part time or menial jobs, in which case they reported an average personal budget of up to € 126.00.

Moreover, it was observed that the most popular smartphones used by respondents (i.e. Techno, Infinix) cost between €54 - 108. In terms of cost of using internet data, it was noticed that a data volume of 1GB per month cost approximately € 2.00; for approximately € 5.00 the respondents said they could get as much as 35GB per month. However, use of mobile data transfer was hampered by low availability of bandwidth and poor internet connectivity.

## Discussion

This feasibility study was conducted several months before the outbreak of COVID-19 but the findings have demonstrated that proactive and novel innovations such as e-learning and m-learning solutions have the potential to enhance the preparedness of health educational institutions towards emergencies such as the COVID-19 global pandemic. As medical education evolves, there is the need for developing countries such as Ghana to adapt to emerging trends in technological innovations. This technological adaption will help improve on the quality of pre-service training for upcoming healthcare professionals. In effect, many more of these feasibility and preparedness studies are highly encouraged in Ghana and Africa at large to inform evidence-based policy decisions on these e-learning and m-learning innovations.

Even though this paper reports mainly on a descriptive data, the findings remain important to local authorities in health institutions of higher learning in Ghana. The focus of this paper at this stage is for local stakeholders of institutions of higher learning who are engaged in the training of healthcare professionals. The expectation is that these results will provide first-hand feasibility and preparedness information to inform design and implementation of future e-learning interventions to promote quality pre-service training for healthcare professionals in Ghana. Lessons learnt from this local context could then be leveraged by other countries within and outside Africa with similar conditions.

Overall, findings of this study suggest that a large percentage of respondents used a wide range of smartphones and applications for staying in touch with friends and getting things done in almost all spheres of life. The smartphone adoption rate in this study reflects the situation reported in previous studies on Ghana and other countries in developing settings [24–26].

It was also discovered that the use of smartphones for education-related activities was already widespread among nursing and midwifery students who participated in the study. This observation corroborates conclusions by previous studies on the topic [24–26]. These revelations appear to suggest frontline health trainees are apparently ready in terms of existing behavioral adjustment to the fast-growing digital revolution catching up with many countries across the globe including countries with less endowed digital infrastructure such as Ghana [27]. The findings also revealed that majority of respondents had the motivation as well as the physical access required to use smartphones, and that they already used a range of applications for purposes related to their learning activities. These findings thus suggest preparedness of these health trainees for m-learning and e-learning innovations or pilot projects.

Another important revelation was the amount of money the health trainees spent on their phones. The self-reported figures suggest expenditure on mobile phones, especially smartphones, makes up a substantial part of their total expenses on and off campus. However, since these figures were mainly self-reported without an independent verification, they should be interpreted with caution. The results generally point to the high utility of mobile phones, particularly, smartphones. Nonetheless, the high connectivity cost was found to be huge bottleneck to the health trainees. The findings thus suggest that even though the respondents appeared to be prepared in terms of high smartphone utilization and being already internet savvy, a potential implementation threat of an e-learning innovation pilot project among these beneficiaries will be the high cost associated with internet connectivity.

Another outcome of this preparedness and feasibility survey was that low availability of bandwidth posed a threat to sustainable implementation of an e-learning innovation pilot even though the findings showed health trainees’ readiness in terms of behavior and acceptability of such intervention. In previous related studies [12, 28, 29] it was observed that even though network coverage (including 3G for mobile data services) was generally satisfactory in many communities of Ghana and other developing countries, the quality of access to the internet in terms of speed of data transfer was insufficient. Findings of this study thus corroborate these earlier conclusions.

On the whole, albeit the average per capita expenditure on mobile phones for general and learning purposes was investigated, the figures quoted by respondents were not independently verified in this study. In light of this, the researchers are unable to rely on these self-reported subjective estimates. According to data from the latest Ghana Living Standards Report [30, 31], the mean annual per capita expenditure in the Volta Region was approximately € 451.08 in 2014. This assumption is based on the estimation that these study participants in the Volta region (the study setting) spent an average of € 4.50 of their disposable income per month on mobile charges (i.e. € 53.96 per year) and that their spending budget is equal to the regional average where students would spend more than 10% of their total expenditure on mobile charges.

The relatively high expenditure of respondents on communications including mobile phone services unfortunately does not commensurate the quality of internet and telecommunication services rendered to subscribers, largely due to poor ICT infrastructure. For instance, an empirical study by Alhassan et al [32] among health tutors in selected nursing and midwifery training schools in Ghana found, limited ICT infrastructure is not only a source of frustration to students and tutors but also an important barrier to quality teaching and learning in these training institutions. Similar studies on the use of mobile technology by health trainees [33–37] and qualified personnel in clinical practice [32, 36–41] revealed that even though the technology is highly beneficial there are constraints regarding its use especially lack of seamless internet connectivity and adaptation by relatively older cadre of health trainees. Findings of this feasibility and preparedness study offers the opportunity to leverage the high mobile phone penetration rate to improve on the quality of pre-service training for healthcare professionals through mobile and e-learning innovations.

### Policy implications and action

Even though the findings are largely descriptive and locally situated, they remain relevant to institutions of higher learning and health sector policy makers. Thus, the following policy recommendations are proposed for institutions of higher learning in Ghana and countries with similar conditions:

1. Health training institutions should initiate policy dialogues on developing e-learning statutory policies to serve as the framework for rollout of e-learning programmes to make these institutions of higher learning globally competitive. Outbreak of COVID-19 has taught institutions of higher learning that e-learning can be leveraged to respond effectively to global pandemics and minimise their effect on academic calendars of these institutions
2. Discussions should be initiated between major telecommunication companies in Ghana and the health training institutions of higher learning, in the spirit of public-private-partnership (PPP), to co-create dedicated internet packages for students and faculty to promote the e-learning agenda. This arrangement could help reduce the per capita expenditure on telecommunication and internet services by beneficiaries and stimulate better utilization of e-learning services.
3. Capacity building workshops and trainings should be institutionalized for students and faculty on emerging trends in e-learning to enhance interest and participation in this digital innovation
4. Finally, health training institutions of higher should create and ring-fence budgets for e-learning to help improve on existing ICT infrastructure and the human resource capacity to promote sustainability of e-learning innovations that are also resource intensive.

### Limitations

Limitations associated with this study are hereby acknowledged. First, the paper reports on a feasibility study that may be largely useful for local stakeholders engaged in pre-service training of healthcare professionals. Unfortunately the findings might not be applicable or generalizable to other settings outside Ghana due to country specific peculiarities. Secondly, perhaps the respondents who consented to participate in the study might have induced self-selection since majority of the participants were within the youthful ages, owned smartphones and hence more likely to be ICT savvy.

Also, the reported results are mainly descriptive to demonstrate the key preparedness and feasibility opportunities and threats to implementing e-learning innovations in the study setting. No rigorous statistical analyses were conducted at this stage. Likewise, the study was conducted in only one public institution out of over 20 institutions in Ghana. Likewise, only one out of the 16 administrative regions in Ghana was selected for this study which posed generalizability challenges.

Moreover, social desirability bias is another potential limitation since some of the self-reported responses were not independently verified by the researchers. Perhaps some participants responded based on so called “favorable responses” to impress the researchers. Nonetheless, given the rigorous adherence to internal validity procedures, the tool remains trustworthy and valid.

The findings of this study remain relevant to informing the policy directions on innovative strategies for pre-service training of health trainees in Ghana and other countries with similar conditions. Nonetheless, it is recommended that future research endeavours should consider conducting full psychometric testing of the developed questionnaire to enhance the reliability and validity of the scale used in this current questionnaire.

## Conclusion

On the whole, there was high prevalence of mobile phone ownership among respondents and high level of preparedness for an e-learning intervention in terms of behavioral adaptability. It was especially observed that the use of smartphones for purposes of learning and exchange of academic information among peers was already an established practice among the study participants. Nonetheless, the main constraints were the low availability of bandwidth and lack of seamless internet connectivity across all telecommunication companies. Even though the average per capita expenditure on usage of the mobile phone was above the European average of 2.5%, subscribers unfortunately appeared not to get value for their money due to the poor quality of telecommunication and ICT services. This constraint is undoubtedly a threat to successful implementation of a future e-learning pilot project.

It is concluded that mobile telephony/ICT space is a huge potential waiting to be fully tapped for effective pre-service training of health trainees in Ghana. Even though there is high preparedness and feasibility of an e-learning pilot project in the study setting, policy implementers must recognize and address the potential threats of high service cost, poor internet connectivity and inadequate ICT infrastructure to make an e-learning project successful and sustainable.

## Data Availability

There is no restriction to data availability. Data is available upon request.

## Abbreviations

HITA: Healthcare Information Technology for Africa
ICT: Information Communication Technology
PPP: Public-private-partnership
REC: Research Ethics Committee
SONAM: School of Nursing and Midwifery
SSA: Sub-Saharan Africa
UHAS: University of Health and Allied Sciences
WHO: World Health Organization

## Footnotes

1 Exchange rate used is based on the prevailing rate at the time of conducting this study (i.e. 1 € = GHC 5.56, source: https://www1.oanda.com/currency/converter/ (27/08/2017))

## References

[1] Okae P (2018). A qualitative study of smartphone usage patterns: the case of Ghana. Science World J.

[2] Omondi G. (2020). GSMA. The state of mobile in Ghana’s tech ecosystem: Ghana’s mobile market: the key trends shaping Ghana’s digital landscape, ecosystem accelerator, 11 February, 2020. Accessed from: https://www.gsma.com/mobilefordevelopment/blog/the-state-of-mobile-in-ghanas-tech-ecosystem/#:~:text=As%20of%20the%20third%20quarter,(as%20of%20Q3%202019) on 7 Oct 2020.

[3] Kinfu Y, Dal Poz MR, Mercer H, Evans DB (2009). The health worker shortage in Africa: are enough physicians and nurses being trained?. Bull World Health Organ.

[4] Sidibé M, Campbell J (2015). Reversing a global health workforce crisis. Bull World Health Organ.

[5] WHO (2016). Global strategic directions for strengthening nursing and midwifery 2016–2020.

[6] World Health Organization (WHO) & Global Health Workforce Alliance (2008). Scaling up, saving lives - task force for scaling up education and training for health workers.

[7] Ministry of Health (MoH) (2014). Health Sector Medium Term Development Plan 2014-2017.

[8] Trump AR, Carr C (2014). Assessing the feasibility of utilizing eLearning content in midwifery schools in Ghana.

[9] Saleh K (2013). The health sector in Ghana – a comprehensive assessment.

[10] Hannafin MJ, Hannafin KM (2010). Cognition and student-centered, web-based learning: issues and implications for research and theory.

[11] Herreid CF, Schiller NA (2013). Case studies and the flipped classroom. J Coll Sci Teach.

[12] Kala S, Isaramalai SA, Pohthong A (2010). Electronic learning and constructivism: a model for nursing education. Nurse Educ Today.

[13] Young LE, Paterson, B. L. (Eds.). Teaching nursing: developing a student-centered learning environment: Lippincott Williams & Wilkins; 2007.

[14] Bell SS (2013). An analysis of nursing education in Ghana: priorities for scaling-up the nursing workforce. Nursing Health Science.

[15] Harandi SR (2015). Effects of e-learning on students’ motivation. Procedia - Social and Behavioral Sciences.

[16] Safie N, Aljunid S (2013). E-learning initiative capacity building for healthcare workforce of developing countries. J Comput Sci.

[17] Harerimana A (2016). Learning in nursing education in Rwanda: benefits and challenges. An exploration of participants’ Perceptives. IOSR Journal of Nursing and Health Science.

[18] Dillenbourg P (1999). Collaborative learning: cognitive and computational approaches. Advances in learning and instruction series.

[19] Murray JP (2011). Educating health professionals in low-resource countries – a global approach.

[20] Ghana Statistical Service (GSS) (2014). Population and housing census (PHC), 2010.

[21] Krejcie KV, Morgan DW (1970). Determining sample size for research activities. Educ Psychol Meas.

[22] Chun Tie Y, Birks M, Francis K (2019). Grounded theory research: a design framework for novice researchers. SAGE open medicine.

[23] Bleda MJ, Tobias A. Cronbach's alpha one-sided confidence interval. Stata Tech Bull. 2001;10(56).

[24] Lwoga E (2012). Making learning and web 2.0 technologies work for higher learning institutions in Africa. Campus-Wide Information Systems.

[25] Feng JY, Chang YT, Chang HY, Erdley WS, Lin CH, Chang YJ (2013). Systematic review of effectiveness of situated e-learning on medical and nursing education. Worldviews Evid-Based Nurs.

[26] McKinney AA, Page K (2009). Podcasts and video streaming: useful tools to facilitate learning of pathophysiology in undergraduate nurse education?. Nurse Educ Pract.

[27] Republic of Ghana. The Ghana ICT for Accelerated Development (ICT4AD). Policy a Policy Statement for the Realization of the Vision to Transform Ghana into an Information-Rich Knowledge-Based Society and Economy through the Development, Deployment and Exploitation of ICTS within the Economy and Society. June, 2003. Accessed from: https://moc.gov.gh/sites/default/files/downloads/Ghana-ICTAD%20Policy-Master-final-2.pdf on 07 Oct 2020.

[28] Holly C (2009). The case for distance education in nursing. J Online Learn Teach.

[29] Lai HC, Chang CY, Wen-Shiane L, Fan YL, Wu YT (2013). The implementation of mobile learning in outdoor education: application of QR codes. Br J Educ Technol.

[30] Barker K, Omoni G, Wakasiaka S, Watiti J, Mathai M, Lavender T (2013). ‘Moving with the times’ taking a glocal approach: a qualitative study of African student nurse views of e learning. Nurse Educ Today.

[31] Voutilainen A, Saaranen T, Sormunen M (2017). Conventional vs. e-learning in nursing education: a systematic review and meta-analysis. Nurse Educ Today.

[32] Alhassan, R. K, Beyere, C.B., Nketiah-Amponsah, E., Mwini-Nyaledzigbor, P.P. (2017). Perceived needs of health tutors in rural and urban health training institutions in Ghana: implications for health sector staff internal migration control. PLoS One, 12(10): 1–11. 10.1371/journal.pone.0185748 PMC5628878 28982194

[33] DeLeo A, Geraghty S (2018). iMidwife: midwifery students’ use of smartphone technology as a mediated educational tool in clinical environments. Contemp Nurse.

[34] Ota M, Peck B, Porter J (2018). Evaluating a blended online learning model among undergraduate nursing students: a quantitative study. CIN: Computers, Informatics, Nursing.

[35] Rahmati R, Mohebbi Dehnavi Z, Kamali Z, Mohebbi Dehnavi A (2018). The effect of Mobile-based and lecture-based training methods on Midwives' knowledge regarding Management of pre-Eclampsia/Eclampsia. Journal of Midwifery and Reproductive Health.

[36] Mbabazi BP, Ali G, Geoffrey A, Lawrence N (2018). Mobile devices for learning in universities: challenges and effects of usage.

[37] Attenborough J, Abbott S (2018). Leave them to their own devices: healthcare students’ experiences of using a range of mobile devices for learning. International Journal for the Scholarship of Teaching and Learning.

[38] Chang CY, Hwang GJ. Trends in smartphone-supported medical education: a review of journal publications from 2007 to 2016. Knowledge Management & E-Learning: An International Journal. 2018;10(4):389–407.

[39] Michel-Schuldt M, Dayon MB, Klar RT, Subah M, King-Lincoln E, Kpangbala-Flomo C, Broniatowski R. Continuous professional development of Liberia's midwifery workforce—a coordinated multi-stakeholder approach. Midwifery. 2018;62:77–80. 10.1016/j.midw.2018.02.023 29655008

[40] O'Connor S, Andrews T. Smartphones and mobile applications (apps) in clinical nursing education: a student perspective. Nurse Educ Today. 2018;69:172–8. 10.1016/j.nedt.2018.07.013 30096510

[41] Urassa D, Chaya P, Pilot J. Addressing knowledge gaps among nurses in healthcare in Tanzania: use of m-learning platforms in Tanzania. Global J Human Soc Sci. 2018;18(1):54–61.

